# Laboratory Surge Capacity and Pandemic Influenza

**DOI:** 10.3201/eid1601.091741

**Published:** 2010-01

**Authors:** Martin I. Meltzer, K. Mills McNeill, Joseph D. Miller

**Affiliations:** Associate Editor, Emerging Infectious Diseases journal (M.I. Meltzer, K.M. McNeill); Centers for Disease Control and Prevention, Atlanta, Georgia, USA (M.I. Meltzer, J.D. Miller); Centers for Disease Control and Prevention, Kampala, Uganda (K.M. McNeill)

**Keywords:** Laboratory, testing, capacity, pandemic influenza, influenza, virus, public health, commentary

In this issue, Crawford et al. describe their experiences running a clinical diagnostic laboratory during the first 3 weeks of the influenza A pandemic (H1N1) 2009 outbreak (*1*). During the early weeks of the outbreak, their laboratory, which serves 15 hospitals and affiliated physician practices in the greater New York City metropolitan area, experienced an ≈8× increase in respiratory virus testing, reaching a maximum of about 900 samples processed in 1 day.

As part of their outbreak response, the laboratory increased weekly work hours by ≈60% and doubled weekend work hours. Physical laboratory space was also rapidly expanded. Equally important to the response plan were 2 decisions to alter testing protocols: cultures were screened 1 time rather than 3, and the use of the Luminex xTAG Respiratory Virus Panel assay (Luminex Molecular Diagnostics, Toronto, Ontario, Canada) was prioritized for testing specimens from hospitalized patients.

The missions of clinical laboratories and public health laboratories (PHL) differ markedly. Clinical laboratories have the primary (almost sole) responsibility of testing samples to aid clinical decision-making. Although PHLs also test samples to aid clinical decisions, functions like surveillance, strain identification, and tracking of drug resistance are arguably their main priorities. Clinical laboratories often have resources available that allow for rapid expansion, but PHLs typically work on fixed budgets that have little flexibility despite unpredictable changes in demand for services.

In their article, Crawford et al. (*1*) discuss many lessons they learned that have universal application for all laboratories engaged in influenza surge response planning. First and foremost was that they had an established plan to deal with such an emergency. Equally important, the laboratory leadership understood the plan and how to adapt it to the specific situation at hand. The leadership also was willing to prioritize testing and triage the flow of samples. The laboratory’s ability to adapt rapidly was limited most notably by the number of suitably trained and experienced staff who could be brought in to provide surge capacity assistance. To be useful, emergency plans must be more than mere documents; they must be rooted in an adequate assessment of capacity and a realistic understanding of the degree to which capacity can be increased rapidly.

The Centers for Disease Control and Prevention has developed a software tool called FluLabSurge (http://www.cdc.gov/flu/tools/flulabsurge), which is designed to assist laboratory directors in planning for a surge in demand for testing. Each laboratory has unique operating characteristics. However, by using FluLabSurge, we determined that the availability of suitably trained laboratory staff is probably the factor that most affects the ability of PHLs to rapidly expand capacity. Thus, public health officials must quickly impose appropriate triage systems at the beginning of public health events, such as an influenza pandemic, to ensure that existing PHL capacity is used effectively and wisely.

Perhaps the most important lessons in the article by Crawford et al. are 1) the need to continually communicate to all clients and stakeholders the need for triaging the flow of clinical samples and 2) the need to explain how testing priorities may change over the course of a pandemic. Such enhanced communication, which clearly explains the limitations of existing laboratory capacity, may help build a constituency that will aid future expansions of PHL capacity.
